# Carbohydrate restriction and dietary cholesterol modulate the expression of HMG-CoA reductase and the LDL receptor in mononuclear cells from adult men

**DOI:** 10.1186/1476-511X-6-34

**Published:** 2007-11-28

**Authors:** Gisella Mutungi, Moises Torres-Gonzalez, Mary M McGrane, Jeff S Volek, Maria Luz Fernandez

**Affiliations:** 1 Department of Nutritional Sciences, University of Connecticut, Storrs, CT, 06269 USA; 2 Department of Kinesiology, University of Connecticut, Storrs, CT 06269, USA

## Abstract

The liver is responsible for controlling cholesterol homeostasis in the body. HMG-CoA reductase and the LDL receptor (LDL-r) are involved in this regulation and are also ubiquitously expressed in all major tissues. We have previously shown in guinea pigs that there is a correlation in gene expression of HMG-CoA reductase and the LDL-r between liver and mononuclear cells. The present study evaluated human mononuclear cells as a surrogate for hepatic expression of these genes. The purpose was to evaluate the effect of dietary carbohydrate restriction with low and high cholesterol content on HMG-CoA reductase and LDL-r mRNA expression in mononuclear cells. All subjects were counseled to consume a carbohydrate restricted diet with 10–15% energy from carbohydrate, 30–35% energy from protein and 55–60% energy from fat. Subjects were randomly assigned to either EGG (640 mg/d additional dietary cholesterol) or SUB groups [equivalent amount of egg substitute (0 dietary cholesterol contributions) per day] for 12 weeks. At the end of the intervention, there were no changes in plasma total or LDL cholesterol (LDL-C) compared to baseline (P > 0.10) or differences in plasma total or LDL-C between groups. The mRNA abundance for HMG-CoA reductase and LDL-r were measured in mononuclear cells using real time PCR. The EGG group showed a significant decrease in HMG-CoA reductase mRNA (1.98 ± 1.26 to 1.32 ± 0.92 arbitrary units P < 0.05) while an increase was observed for the SUB group (1.13 ± 0.52 to 1.69 ± 1.61 arbitrary units P < 0.05). Additionally, the LDL-r mRNA abundance was decreased in the EGG group (1.72 ± 0.69 to 1.24 ± 0.55 arbitrary units P < 0.05) and significantly increased in the SUB group (1.00 ± 0.60 to 1.67 ± 1.94 arbitrary units P < 0.05). The findings indicate that dietary cholesterol during a weight loss intervention alters the expression of genes regulating cholesterol homeostasis.

## Background

Cholesterol is an important biological molecule that plays a role in membrane structure and it is a precursor for the synthesis of steroid hormones and bile acids [1,2]. It is also a major player in the lipid rafts which have a vital role in cell signaling and protein sorting on the membrane surface [3-5]. Mammalian cells produce their own cholesterol and receive cholesterol by uptake from lipoproteins [5]. Regulation of synthesis, influx and efflux keeps cellular cholesterol levels precisely controlled [5]. The synthesis of cholesterol is regulated by the activity of the microsomal enzyme 3-hydroxy-3 methylglutaryl coenzyme A (HMG-CoA) reductase, and this is determined by both the amount of protein present and the degree of activation of the enzyme (dephosphorylation) [6,7]. One of the main mechanisms of cholesterol uptake by the cells is through the LDL receptor (LDL-r). The LDL-r synthesis is activated by low concentrations of cellular free cholesterol [8]. Animal data suggest that cholesterol synthesis and cholesterol uptake by the LDL-r may work independently, and that LDL uptake by the receptor is a secondary compensatory mechanism after cholesterol synthesis [9]. Cholesterol homeostasis is the mechanism of ensuring appropriate physiological cholesterol levels [5,10]. First demonstrated in vitro, the LDL-r gene is down regulated by higher intracellular cholesterol [10,11] and so is HMG-CoA reductase [12]. Dietary macronutrients have a major impact on plasma lipids, and both LDL-r and HMG-CoA reductase have a central role in lipid metabolism [13]. Therefore we determined the mRNA expression of these two genes in response to two levels of dietary cholesterol in the context of a low carbohydrate intake. We demonstrated that increased intake of cholesterol affects mRNA expression of both HMG-CoA reductase and LDL-r.

## Methods

### Diets

These were free living subjects who were not provided with any other foods apart from either eggs or eggs substitute to consume as part of a low carbohydrate diet. No restrictions were given towards energy intake. Subjects received individual and personalized dietary counseling from Registered Dietitians prior to the dietary intervention. Detailed dietary booklets, specific to each dietary treatment, were provided outlining dietary goals, lists of appropriate foods, recipes, sample meal plans, and food record log sheets. Subjects received weekly follow-up counseling during which body mass was measured, compliance was assessed, and further dietetic education was provided. A three-day weighed food records was obtained at baseline to assess habitual nutrient intake, and a five-day records were completed during weeks 1, 6, and 12 of the intervention.

Subjects were given specific instructions on how to follow a carbohydrate restricted diet (CRD), as previously reported from our lab [14]. Subjects were asked to maintain their normal routine of physical activities during the course of this study.

### Mononuclear cell Isolation

Whole blood was used to isolate mononuclear cells following method by Boyum [15]. In this method 20 mL blood was diluted with 10 mL HBSS (Sigma-Aldrich) that did not contain either Ca^2+ ^or Mg^2+^. Then this diluted solution was carefully layered over 10 mL of Histopaque 1077 (Sigma-Aldrich), and centrifuged at 500 × g for 30 minutes (Rotanta 460 R). The mononuclear cell interface was removed and washed with HBSS and centrifuged at 600 × g for 10 minutes twice. The cell pellet was resuspended in 200 μL Tris buffer (150 mmol/L NaCl, 10 mmol/L Tris, 1 mmol/L CaCl2, pH 7.4), and the sample was stored at -80°C.

### RNA extraction and purification

Total RNA was extracted from mononuclear cells using a slightly modification of the method of Chomczynski and Sacchi [16]. TRIzol reagents were used as per manufacturer's guidelines. The integrity of the extracted RNA was checked by electrophoresis on a 1% agarose gel. The DNA-free kit was used to remove trace contaminating genomic DNA following the manufacturer's instruction (Bio-Rad). RNA was extracted by precipitation using 2.5 volumes of 100% ethanol and 0.1 volume of 3 mol/L sodium acetate at pH 5.2. The RNA pellet was washed with 70% ethanol and dissolved in diethyl pyrocarbonate-treated water. cDNA synthesis was done using iScript cDNA synthesis kit as per manufacturer's instructions (Bio-Rad).

### Real-time PCR

LightCyler Probe Design software 2.0 (Roche Diagnostics) was used to design primers for the target genes, HMG-CoA reductase, LDL-r and the reference gene glyceraldehyde-3-phosphate dehydrogenase (GAPDH) [17].

Using the LightCycler FastStart DNA Master plus SYBR Greeen I (Roche Diagnostics) real-time Polymerase Chain Reaction (PCR) was performed in duplicate using the LightCycler 2.0. A melting curve was obtained after amplification, to determine the optimal PCR conditions. By analyzing the fluorescence curves and detecting the crossing point of samples using LightCycler software 4.0 (Roche Diagnostics) quantification of mRNA was done.

### Plasma Lipids

Total cholesterol (TC) and HDL cholesterol (HDL-C) were determined by enzymatic methods. LDL Cholesterol (LDL-C) was calculated by the Friedwal equation after measurement of plasma total cholesterol, triglycerides and HDL-C as previously reported [18].

### Statistical analysis

Data was analyzed using Repeated Measures ANOVA with time being the repeated measure and EGG versus SUB the between subjects factor. Data are presented as means ± SD. Differences of *P *< 0.05 were considered significant.

## Results

Plasma TC was not affected by diet or over time. Values were 198.3 ± 42.1 mg/dL at baseline and 202.2 ± 41.8 mg/dL at 12 wk for the EGG group and 188.3 ± 33.7 and 187.3 ± 39.5 at baseline and 12 wk respectively for the SUB group. In spite of the greater consumption of dietary cholesterol in the EGG group (877 mg/d) at wk 12 compared to the SUB group (277 mg/d), there were no increases in LDL-C between baseline and 12 wk or differences in LDL-C between the EGG and the SUB groups. Values were 127.5 ± 42.2 mg/dL at baseline and 144.3 ± 45.1 mg/dL after 12 wk for the EGG group while values for the SUB group were 110.8 ± 34.5 mg/dL for baseline and 121.5 ± 42.2 mg/dL after 12 wk (P > 0.1). In contrast, HDL-C was significantly increased only in the EGG group from 47.6 ± 15.1 mg/dL at baseline to 57.1 ± 15.1 mg/dL at 12 wk while there were no changes in HDL-C in subjects from the SUB group (50.0 ± 9.7 mg/dL at baseline and 48.8 ± 8.8 mg/dL after 12 wk).

**Figure 1:**
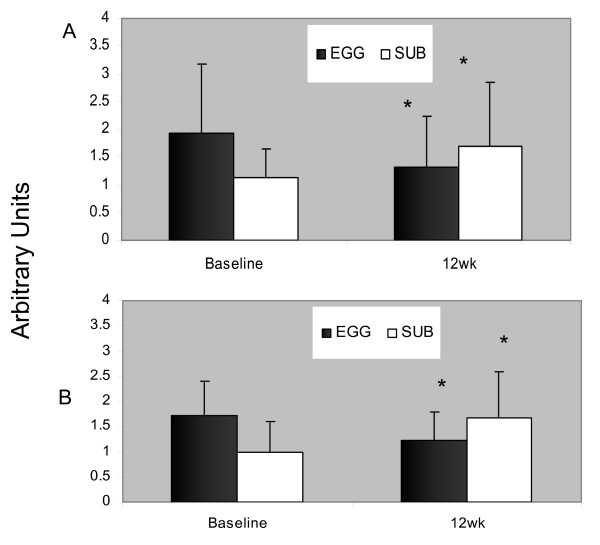
HMG-CoA reductase mRNA abundance in mononuclear cells isolated from subjects from the EGG (n = 14) and SUB (n = 11) groups (Panel A); LDL receptor mRNA abundance in mononuclear cells isolated from subjects from the EGG (n = 15) and SUB (n = 13) groups. Values represent arbitrary unit presented as means ± standard deviation. * indicates significantly different from baseline.

Results for gene expression at baseline and 12 wk are indicated in Figure 1 for the EGG and the SUB groups. HMG-CoA reductase gene expression was down-regulated by 31% and the LDL-r by 29% in the EGG group (P < 0.05) while in the SUB group the expression of both genes was up-regulated by 53 and 66% respectively (P < 0.05).

## Discussion

Mononuclear cells were used as a surrogate to reflect hepatic cholesterol homeostasis in response to a cholesterol challenge. The present study suggests the involvement of dietary cholesterol in gene regulation. In the EGG group there was a 298% increase in dietary cholesterol intake compared to the SUB group. The LDL-C did not change during the intervention for both the EGG and SUB groups. The increase in dietary cholesterol on the EGG group might have contributed to the increase in intracellular cholesterol and significantly down regulate the mRNA expression of LDL-r and HMG-CoA reductase. These findings are supported by Meddings et al.,[19] and Lin et al., [20]. High dietary cholesterol generally elevates serum LDL-C, which might result in an increase in intracellular cholesterol via cholesterol uptake. The failure to observe the increase in plasma LDL cholesterol in the present study can be explained by the weight reduction, which might have prevented an increase accumulation of LDL-C. Studies in guinea pigs have shown that dietary cholesterol increases hepatic tissue cholesterol [21], which could possibly explain the down regulation of HMG-CoA reductase in the current work.

Studies using cultured cells [10], showed that a reduction in intracellular cholesterol levels resulted in a dual response: 1. an increase in the production of mRNA for HMG-CoA reductase to synthesize more cholesterol for the depleted cell and 2. an increase in the amount of mRNA for the LDL-r in order to increase exogenous cholesterol uptake.

A previous study in our lab in pre-menopausal women has shown that weight loss results in the increase of LDL-r mRNA abundance [22]. A study using the fat Zucker rat [23], showed that these rats had reduced expression of hepatic LDL-r as compared to lean ones. A dietary cholesterol challenge had a suppressive outcome on hepatic LDL-r in both the Zucker and control rats. How does obesity influence cholesterol status? In obesity where there is no cholesterol challenge, the tissue might have a sufficient supply of cholesterol as a result of stored cellular lipids, and hence, the down regulation of the mechanism involved in the increase of cellular cholesterol synthesis and uptake. With weight loss where most of the weight reduced is from adipose tissues, there is a depletion of stored and available cellular cholesterol, and therefore, the mechanism to increase cellular cholesterol is up-regulated. This study supports these findings since the SUB group significantly increased LDL-r mRNA as a result of weight loss. Additionally, the findings for the EGG group suggests that the presence of a cholesterol challenge on a weight loss overrides the effect of LDL-r mRNA expression during weight loss, and this results in down regulation of the LDL receptor.

## Abbreviations

CRD: carbohydrate restricted diets; HDL-C: HDL cholesterol; HMG-CoA: 3-hydroxy-3methyl glutaryl Coenzyme A; LDL-C: LDL cholesterol; LDL-r: LDL receptor; TC: total cholesterol.

## Competing interests

The author(s) declare that they have no competing interests.

## Authors' contributions

All authors read and approved the final manuscript. GM did the assays, wrote the manuscript and participated in the interpretation of data; MT: assisted in data collection, interpretation and critical evaluation of manuscript; MM: developed the molecular techniques and assisted in data interpretation; JSV participated with experimental design and critical evaluation of the data and MLF designed the experiment, evaluated the results, interpreted the data and participated in manuscript preparation.
